# Graphene-Based Nanomaterials Modulate Internal Biofilm Interactions and Microbial Diversity

**DOI:** 10.3389/fmicb.2021.623853

**Published:** 2021-03-26

**Authors:** Lauris Evariste, Paul Braylé, Florence Mouchet, Jérôme Silvestre, Laury Gauthier, Emmanuel Flahaut, Eric Pinelli, Maialen Barret

**Affiliations:** ^1^Laboratoire d’écologie fonctionnelle et environnement, Université de Toulouse, CNRS, INPT, UPS, Toulouse, France; ^2^CIRIMAT, Université de Toulouse, CNRS, INPT, UPS, UMR CNRS-UPS-INP N°5085, Université Toulouse 3 Paul Sabatier, Bât. CIRIMAT, Toulouse, France

**Keywords:** microbial ecotoxicology, graphene, freshwater, biofilm, diatom, metabarcoding

## Abstract

Graphene-based nanomaterials (GBMs), such as graphene oxide (GO) and reduced graphene oxide (rGO), possess unique properties triggering high expectations for the development of new technological applications and are forecasted to be produced at industrial-scale. This raises the question of potential adverse outcomes on living organisms and especially toward microorganisms constituting the basis of the trophic chain in ecosystems. However, investigations on GBMs toxicity were performed on various microorganisms using single species that are helpful to determine toxicity mechanisms but fail to predict the consequences of the observed effects at a larger organization scale. Thus, this study focuses on the ecotoxicological assessment of GO and rGO toward a biofilm composed of the diatom *Nitzschia palea* associated to a bacterial consortium. After 48 and 144 h of exposure to these GBMs at 0, 0.1, 1, and 10 mg.L^−1^, their effects on the diatom physiology, the structure, and the metabolism of bacterial communities were measured through the use of flow cytometry, 16S amplicon sequencing, and Biolog ecoplates, respectively. The exposure to both of these GBMs stimulated the diatom growth. Besides, GO exerted strong bacterial growth inhibition as from 1 mg.L^−1^, influenced the taxonomic composition of diatom-associated bacterial consortium, and increased transiently the bacterial activity related to carbon cycling, with weak toxicity toward the diatom. On the contrary, rGO was shown to exert a weaker toxicity toward the bacterial consortium, whereas it influenced more strongly the diatom physiology. When compared to the results from the literature using single species tests, our study suggests that diatoms benefited from diatom-bacteria interactions and that the biofilm was able to maintain or recover its carbon-related metabolic activities when exposed to GBMs.

## Introduction

Two-dimensional nanomaterials derived from graphene possess unique properties such as high surface area, electrical and thermal conductivity, mechanical strength, and optical transmittance that are currently being explored for the development of new applications in multiple area including composite improvement, energy storage, electronics, medicine, or water purification (Perreault et al., 2015; Dasari Shareena et al., 2018; Mohan et al., 2018; Nag et al., 2018). Among these graphene-based nanomaterials (GBMs), graphene oxide (GO), and reduced graphene oxide (rGO) appear as very attractive due to their ease of synthesis, their high stability after dispersion in various solvents and the possibility for surface functionalization (Smith et al., 2019). rGO, which carries a lower amount of oxygen-containing functions compared to GO (Lavin-Lopez et al., 2017), constitutes a good compromise between GO and graphene, especially for electrical conductivity properties, leading to its use for the development of electrochemical sensors (Rowley-Neale et al., 2018; Tarcan et al., 2020). As the production of high quality graphene suffers from high energy consumption and cost, GO and rGO constitute major products in the graphene market (Lin et al., 2019). For these reasons, these GBMs are forecasted to be mass-produced and are thus likely to be released in the environment during their whole life cycle, from the production to the recycling (Mottier et al., 2017). However, the monitoring of environmental pollutions by GBMs is not possible yet due to technical limitations for their detection at current low concentrations in complex matrices (Goodwin et al., 2018). Nevertheless, despite the lack of modeling data about their expected environmental concentrations, it is estimated that with increasing needs, GBMs could reach concentrations between 1 and 1,000 μg/L in aqueous environment (Zhang et al., 2017), with an accumulation trend in sediment (Sun et al., 2016; Avant et al., 2019). This requires to carefully evaluate the potential impact of these materials on environmental health, in order to contribute to the development of this nanotechnology in a safety and sustainable way (Fadeel et al., 2018).

Previous studies investigated the toxicity of GBMs toward various aquatic organisms including vertebrates (Clemente et al., 2019; Evariste et al., 2019; Paital et al., 2019) or invertebrates (Castro et al., 2018; Lv et al., 2018), while the most abundant literature concerns the effects on bacteria and microalgae (Han et al., 2019; Kumar et al., 2019; Tashan et al., 2019; Saxena et al., 2020). Studying the effects of GBMs on these microorganisms is essential since they play crucial roles in aquatic ecosystems. Indeed, microalgae ensure primary production through photosynthesis while bacterial heterotrophic activities contribute to organic matter and nutrient cycling (Paerl and Pinckney, 1996; Scala and Bowler, 2001). Moreover, these microorganisms are at the basis of the trophic chain in the environment and act as a resource supplier for many primary consumers. Thus, impairment of these communities by GBMs exposure could indirectly affect organisms from higher trophic levels (Evariste et al., 2020). The vast majority of the studies available on bacteria and algae were performed on isolated single strains (e.g., *Escherichia coli*, *Staphylococcus aureus*, and *Chlorella* sp.) and highlighted antibacterial activities, and algal growth inhibitory effects of GBMs that were both associated to oxidative stress and membrane injuries (Ouyang et al., 2015; Ji et al., 2016; Zhao et al., 2017). Although these effects are well-documented in free-living cells, data concerning GBMs toxicity toward microorganisms living in complex biofilms remain scarce and inconsistent despite the fact that environmental biofilms are recognized to bind and accumulate nanoparticles (Ikuma et al., 2015). Biofilm lifestyle confers ecological advantages over free-living cells as it includes social cooperation as well as enhanced resource capture and resistance to antimicrobials for the organisms embedded in a matrix of extracellular polymeric substances (EPS; Flemming et al., 2016).

The main literature available focus on the effects of metallic nanoparticles toward biofilms, while studies focusing on carbon-based nanomaterials remains scarce (González et al., 2015; Lawrence et al., 2016; Hou et al., 2017; Miao et al., 2019). Specifically, the effects of GBMs toward biofilms were monitored on single bacterial strains in order for the development of antimicrobial treatments to avoid the formation of pathogenic biofilms for biomedical purposes (Han et al., 2019; Liu et al., 2019; Cacaci et al., 2020; Cao et al., 2021). In this context, studies indicated that GO-coated surfaces could either promote or inhibit biofilm formation by *E. coli* and *S. aureus* (Ruiz et al., 2011; Guo et al., 2017; Yadav et al., 2017). However, these studies were performed under conditions that are not fully relevant within environmental-based contexts that are not sufficiently investigated (Jastrzębska and Olszyna, 2015; Montagner et al., 2016). Thus, understanding the toxicological effects of GBMs toward more complex communities is crucial to better characterize the ecotoxic potential of these nanomaterials and to further determine the possible consequences of their presence in freshwater environments on the ecosystem functioning. The aim of this study was to investigate the toxicity of a commercial GO and its reduced form toward a complex assembly composed by the diatom *Nitzschia palea* associated to a bacterial consortium. This biofilm was exposed under controlled conditions to GBMs at concentrations ranging from 0.1 to 10 mg.L^−1^ to determine the effects on diatom physiology using flow cytometry, as well as on bacterial community structure and activity using high throughput 16S sequencing and community-level physiological profiles, respectively.

## Materials and Methods

### Graphene-Based Nanomaterials

Graphene oxide was provided by Antolin Group and prepared by oxidizing Grupo Antolin Carbon Nanofibers (GANF®; Grupo Antolín, Burgos, Spain) using the Hummer’s method (Hummers and Offeman, 1958; Lobato et al., 2016). We thermally reduced it at 200°C in H_2_ atmosphere into rGO, as previously described (Evariste et al., 2019). GBMs were stored as dry powder in the dark and dispersions were prepared extemporaneously in order to avoid any possible change of material characteristics. Full characterization of the tested materials was detailed in previous work (Evariste et al., 2019) and characterization data are summarized in Table 1.

**Table 1:** Physico-chemical characteristics of graphene oxide (GO) and reduced graphene oxide (rGO) used in the study.

	Graphene Oxide	Reduced Graphene Oxide
Carbon content (at. %)	69.0 ± 0.4	83.8 ± 0.5
Oxygen content (at. %)	31.0 ± 0.4	16.2 ± 0.3
Csp2 graphene (at. %)	35.5	64.5
C▬OH/C▬O▬C (at. %)	24.7	7.8
C═O (at. %)	2.5	5.8
O═C▬O (at. %)	5.3	1.3
Sat. (at. %)	1.4	4.5
Number of layers (HRTEM)	1–5	1–5
Lateral size (TEM; μm)	0.2–8	0.2–8
Specific surface area (BET; m^2^.g^−1^)	228 ± 7	16 ± 0.5

TEM, transmission electron microscope; HRTEM, high resolution TEM; BET, Brunauer-Emett-Teller; at. %, atomic %.

### Complex Biofilm Model and Exposure Procedure

The experimental model for complex biofilms was composed of an association between an axenic strain of *N. palea* CPCC-160 provided by the Canadian Phycological Culture Center (University of Waterloo, Waterloo, ON, Canada) and a bacterial consortium isolated from water filters of the freshwater Museum-Aquarium of Nancy (France). After sampling, the consortium was suspended in 50% glycerol and stored at −80°C until use.

Before the beginning of the exposure to GBMs, microorganisms were sequentially introduced in culture Flasks (Falcon 355,001, 600 ml – 150 cm^2^) as follows. *Nitzschia palea* was cultured as previously described in a modified CHU no. 10 basic medium, called SPE medium (SPE; 6.4 < pH < 6.6; Garacci et al., 2017). Standard growth conditions consisted in an incubation at 22 ± 1°C on a rotary shaker (50 rpm) in a culture room. An illumination of 50 μmol m^−2^ s^−1^ with a day/night period of 14/10 h, respectively, was applied. Two days prior to exposure (T_−48h_), diatoms were transferred at a density of 5 × 10^4^ cells/ml in a flask containing 50 ml of SPE medium. After 24 h of growth in standard conditions (T_−24h_), diatoms reached a concentration of 9 × 10^4^ cells/ml. Glycerol was removed from the bacterial consortium after centrifugation and the consortium was suspended in 100 ml of SPE medium. Thus, at T_−24h_, flasks containing diatoms were inoculated with the bacterial consortium to reach the concentration of 3 × 10^4^ bacterial cells/ml, leading to the ratio of 3 diatoms per bacterial cells in each flask. After 24 h, flasks were contaminated with GBMs (T0). For this purpose, nanomaterials were dispersed in SPE medium through the use of an ultrasonic bath for 10 min and autoclaved. Dilutions of the GBMs stock dispersions were carried out under axenic conditions in order to avoid contamination. Intermediary dispersions were prepared at 0.2, 2 and 20 mg.L^−1^. A volume of 50 ml of GBMs-contaminated SPE media was added in the flasks to reach a final concentration of 0.1, 1, or 10 mg.L^−1^ of GBMs while uncontaminated medium was added in the control groups (T0). Exposures were performed over 144 h under standard conditions as previously indicated.

Biofilm was sampled after 48 and 144 h of exposure with the different concentrations of GBMs (*n* = 3 per time and GBM concentration). For each sampling, the biofilm was gently scrapped and flask content was homogenized and divided into two fractions of 50 ml. The first fraction was used for diatom and bacteria counts as well as analysis of the diatom physiological parameters using flow cytometry. In addition, this fraction was also used to perform community level physiological profiles (CLPP) analysis. The second 50 ml fraction was filtrated at 0.45 μm (Whatman® Nuclepore™) to collect the whole microorganism community and investigate the microbial community structure. Filters were stored individually in sterile tubes at −80°C prior to further processing.

### Flow Cytometry Analysis

Flow cytometric (FCM) analysis of the diatom *N. palea* and bacterial counts were performed using a Beckman-Coulter Cytoflex flow cytometer equipped with a 488 nm laser and data were collected and analyzed using Cytexpert v. 2.2.0.97 software.

#### Microorganisms Counts and Growth Rate Calculation

For *N. palea* counts, unstained algae were gated based on their forward scatter parameters (FSC-A) and chlorophyll fluorescence (690/50 nm). Bacteria were accounted using SYTO9 dye (Invitrogen). Samples were incubated with 5 μM of the probe for 15 min in the dark at room temperature. Bacteria were detected and enumerated based on the fluorescence emitted by SYTO9-positive events (525/40 nm) and side scatter parameters (SSC-H). Normalized growth rates of the diatoms and bacteria were calculated as follows:

$$Growthrate=\left(\frac{Cs-Mic}{Mic}\right)$$

$$Normalizedgrowthrate\;\left(\%\right)=\left(\frac{Growthrate}{MCtGr}\right)\;x\;100$$

Cs corresponds to the organism concentration at sampling time, Mic is the mean initial concentration of organisms (at T_−48h_ for diatoms and at T_−24h_ for bacteria) and MCtGr is the mean growth rate in the control group at sampling time.

#### Diatom Physiological Parameters

The relative chlorophyll a content of *N. palea* was determined through the measurement of natural chlorophyll a fluorescence emitted at 690/50 nm. The mean fluorescence intensity (MFI in arbitrary unit) collected is expressed as a percentage of the negative control.

Diatom viability was evaluated using SYTOX® Green. After incubation with the probe for 10 min at a final concentration of 0.5 μM, cell suspensions were analyzed by flow cytometry to measure the fluorescence emitted at 525/40 nm. Diatoms with injured or permeable membrane are positive to the green fluorescence-emitting probe bound to DNA. Results are expressed as viability percentage (100% – percentage of SYTOX-positive cells).

Neutral lipid relative content of the diatoms was evaluated using BODIPY (4,4-difluoro-1,3,5,7,-tetramethyl-4-bora-3a,4a-diaza-s-indacene; 505/515). Algae were incubated for 1.5 min with the lipophilic dye at a final concentration of 1 μg.ml^−1^ before FCM. The BODIPY fluorescence emitted by the stained diatoms was collected using a 530/30 nm band-pass filter. The MFI measured is presented as a percentage of the negative control.

The intracellular reactive oxygen species (ROS) produced by the diatoms was measured using 2′,7′-dicholorofluorescindiacetate (DCFH-DA), a marker of oxidative stress. Samples were stained for 30 min with the probe at a final concentration of 10 μM prior running flow cytometry measurements. Diatoms with elevated intracellular ROS are positively stained by the probe (Thamatrakoln et al., 2012). The results are presented as a percentage of the diatom population emitting probe-related fluorescence.

### Analysis of Community Level Physiological Profiles

BIOLOG® EcoPlates, consisting of 96-well plates containing a triplicate of 31 different carbon sources and a control with no carbon source, were used to analyze the CLPP of the microorganism communities. Samples were diluted 100 times with fresh SPE medium and 120 μl of the diluted suspension was transferred into each well of the plate. After inoculation, EcoPlates were incubated in aerobic conditions at 22 ± 1°C in darkness for 144 h. Optical density (OD) at 590 nm was measured immediately after microorganism plating (T_in_) and was monitored daily over the 144 h with a CLARIOStar plate reader (BMG Labtech). Over these 144 h, OD increased linearly (*r*^2^ = 0.94) and did not reached a plateau phase. For each substrate, absorbance was corrected by subtracting the absorbance of the control well containing media only. Negative values of the corrected readings were set to zero. The average well color development (AWCD) of substrate utilization was calculated across all wells per plate as follows:

$$AWCD=\left(\sum ODi\right)/31,$$

where *ODi* represent the corrected optical density of the ith well. AWCD was also calculated for each guild of carbon sources, grouped into (1) carbohydrates, (2) carboxylic acids, (3) amino acids, (4) amines and amides, and (5) polymers as defined by Weber and Legge (2009). To compensate the influence of the microorganism density on the AWCD measurement, corrected OD values were calculated per bacteria by dividing the OD value per the number of bacteria introduced in each well at T_in_. The normalized carbon source utilization data were also subjected to principal components analysis (PCA). All the results reported refer to the 144 h time point.

### Analysis of Bacterial Community Structure: DNA Extraction, PCR, Sequencing, and Data Processing

After cutting filters into pieces, total DNA was extracted using the QIAGEN DNeasy PowerSoil kit following manufacturer’s instructions. Extraction controls were performed using unused filters to ensure the absence of DNA contamination. The DNA extracts quantity and quality were analyzed using a NanoDrop 2000 UV spectrophotometer (Thermo Scientific). The V4-V5 region of 16S rRNA gene was targeted for *Archaea* and Bacteria using 515F (5'-GTGYAGCMGCCGCGGTA-3') and 928R (5'-CCCCGYCAATTCMTTTRAGT-3') primers set (Wang and Qian, 2009). PCR reactions were run in a final volume of 50 μl containing: 37.5 μl of PCR water, 5 μl of 10X PCR buffer, 2 μl of DNA extract, 2 μl of both primer, 1 μl of dNTP (2.5 mM) and 0.5 μl of Taq DNA polymerase (5 U/μl – Sigma Aldrich). The following PCR protocol was applied: 94°C for 120 s, 30 cycles of 94°C for 60 s, 65°C for 40 s, 72°C 30 s, and 72°C for 10 min. Sequencing of the resulting amplicons was performed using Illumina MiSeq technology (2 × 250 pb) by the Get_PlaGe platform (Genotoul, Toulouse, France). Bioinformatic analysis was performed using Find Rapidly Operational Taxonomic Units Galaxy Solution (FROGS) pipeline on Galaxy (Escudié et al., 2018). Briefly, sequences with mismatch in the primers were excluded and PCR primers were trimmed. Reads were clustered into operational taxonomic units (OTUs) using the Swarm clustering method (Mahé et al., 2014). Chimera was removed and filters were applied to remove singletons and keep OTUs present in at least two samples. OTUs were assigned at different taxonomic levels (from Kingdom to species) using RDP classifier and NCBI Blast+ against Silva 138 database (pintail 80; Quast et al., 2012). Amplicons affiliated to the diatom chloroplast and mitochondria were removed from the dataset prior to data analysis.

### Statistical Analysis

As interactive effects were observed between the exposure duration and the contaminant concentrations using two-ways ANOVA, one-way ANOVA was performed at each sampling time to compare the effects induced by the different concentrations of contaminant. Thus, data related to microorganism growth rates, diatom physiological parameters, and community physiological profiles were analyzed using one-way ANOVA when assumptions of normality and homogeneity of variance were met. Otherwise, data were transformed to meet these assumptions and data were analyzed using Minitab 16 Statistical software. Concentrations leading to 50% of bacterial growth inhibition (IC_50_) were determined using non-linear Hill regression in Graphpad Prism software.

Sequencing data analyses for OTUs counts, alpha diversity indexes, and Weighted Unifrac Distances calculations as well as multidimensional scaling (MDS) plot were carried out using the R package “Phyloseq” (McMurdie and Holmes, 2013). Differential abundance of bacterial genera between exposed conditions compared to the control group was examined using “Deseq2” R package (Love et al., 2014). PERMANOVA was performed using Adonis function from the “vegan” R package (Oksanen et al., 2015).

## Results

### Effects on Microorganism Growth Rates

**Figure 1:**
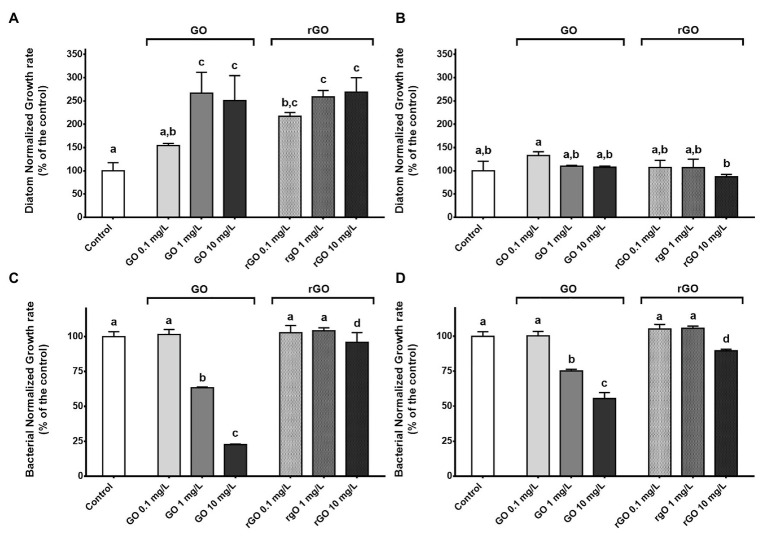
Normalized growth rate of the diatom *Nitzschia palea* calculated after 48 h **(A)** and 144 h **(B)** of exposure to graphene-based nanomaterials (GBMs). Normalized growth rate of the bacterial consortium calculated after 48 h **(C)** and 144 h **(D)** of exposure to GBMs. ANOVA was followed by Tukey test. Letters indicate significant differences between the tested conditions.

Exposure to the GBMs led to a transitory growth stimulation of the diatom. At T_48h_, the growth rate calculated in the control group reached a value of 5.4 ± 0.95 (Supplementary Figure S1). Except after exposure to GO at 0.1 mg.L^−1^, a significant growth stimulation was observed in all GBMs-containing conditions (ANOVA *p* < 0.001), reaching 268 ± 31% of the control group value after exposure to 10 mg.L^−1^ of rGO (Figure 1A). After 144 h of exposure, none of the growth rates calculated in the exposure conditions were significantly different from the control group (Figure 1B). In addition, growth rates of the diatom calculated after 48 h of exposure to GBMs reached values similar to the control group at T_144h_ (12.7 ± 2.6; *t*-test; *p* = 0.604; Supplementary Figure S1).

For the bacterial compartment, exposure to GO led to a dose dependant growth inhibition from 1 mg.L^−1^, while only a slight inhibition was noticed after exposure to rGO at 10 mg.L^−1^ (Figure 1C). The calculated concentrations leading to a growth inhibition of 50% (IC_50_) were 2.18 mg.L^−1^ and 13.25 mg.L^−1^ after 48 and 144 h of exposure to GO, respectively. Contrary to the recovery observed for diatoms at 144 h, bacterial growth rates were still significantly different from the control at 1 and 10 mg.L^−1^ of GO to at 10 mg.L^−1^ of rGO (Figure 1D).

### Effects on *N. palea* Physiology

**Figure 2:**
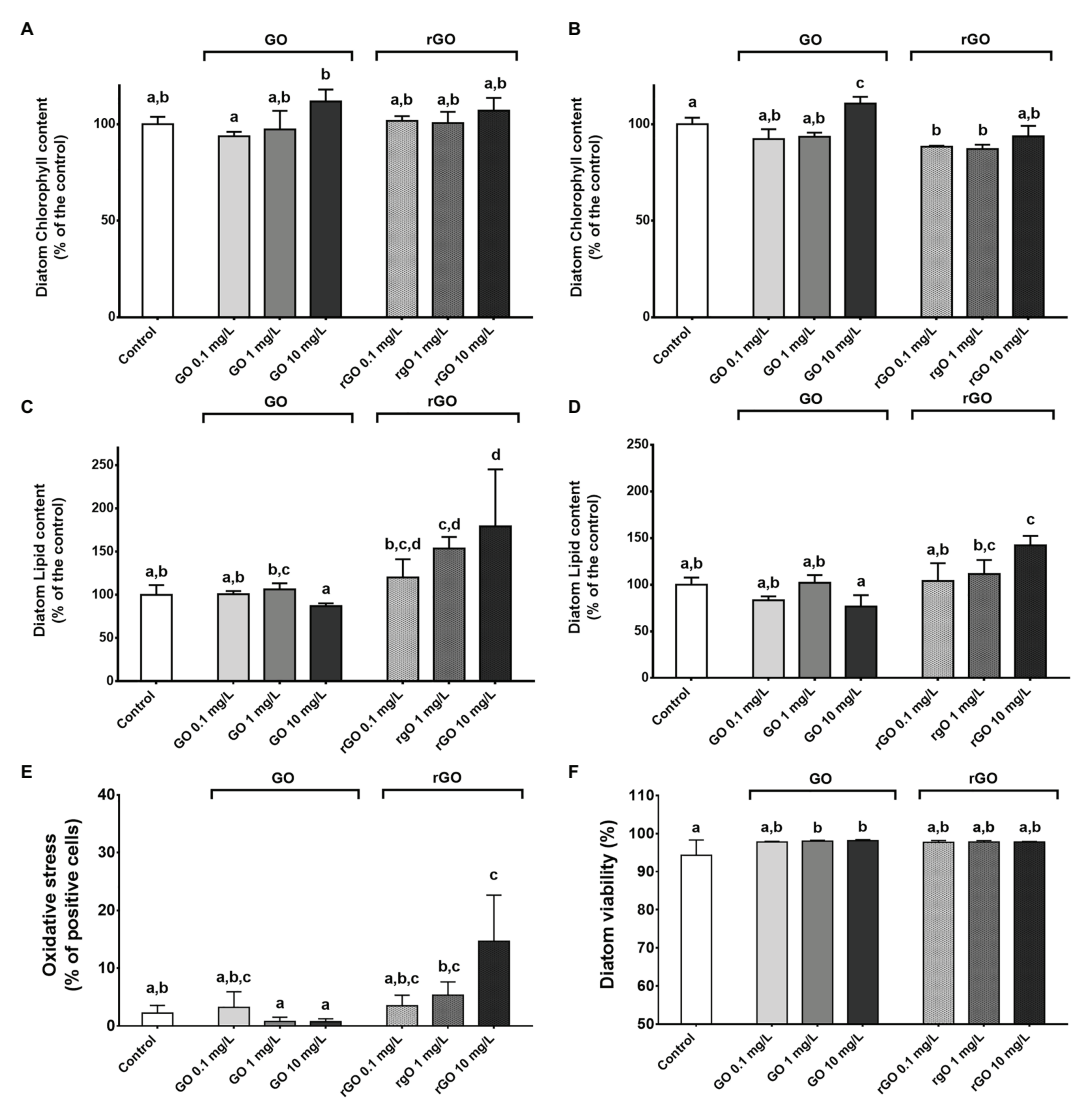
Physiological parameters of the diatom *N. palea* following exposure to GBMs. Normalized chlorophyll content measured after 48 h **(A)** and 144 h of exposure **(B)**, normalized lipid content measured after 48 h **(C)** and 144 h **(D)**, oxidative stress **(E)** and viability **(F)** following 48 h of exposure to GBMs. Values are presented as mean ± SD. ANOVA (*p* < 0.05) was followed by Tukey test. Letters indicate significant differences between the tested conditions.

The relative chlorophyll content of the diatom remained unchanged after 48 h of exposure to the GBMs (Figure 2A) while a higher relative chlorophyll content could be measured in the diatoms exposed to GO at 10 mg.L^−1^ during 144 h (Figure 2B). On the contrary, exposure to rGO led to a slight decrease of the chlorophyll content after 144 h of contact with rGO (Figure 2B).

Graphene oxide exposure did not lead to any significant change in the lipid content of the diatom while in rGO containing conditions, an accumulation of lipids was measured, with a more marked effect after 48 h of exposure (Figures 2C,D).

Measurement of oxidative stress in the diatom indicated a significant oxidative stress after 48 h of exposure to rGO at 10 mg.L^−1^ (Figure 2E) while no DCF-positive cells were observed at T_144h_ (data not shown). Monitoring of the diatom viability highlighted a significantly higher percentage of alive diatoms after 48 h of exposure to GO at 1 and 10 mg.L^−1^ (ANOVA, *p* = 0.027, Figure 2F). At T_144h_, no differences were observed between the different GBMs-containing conditions and the control group in the diatom viability that reached that 99 ± 0.64% (data not shown).

### Effects of GBMs Exposure on Community Level Physiological Profiles

**Figure 3:**
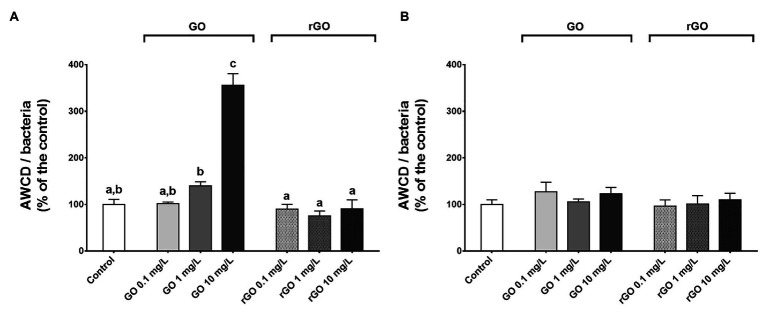
Normalized average well color development (AWCD) values measured after 48 h **(A)** and 144 h **(B)** of exposure to GBMs. ANOVA followed by Tukey test. Letters indicate significant differences between the tested conditions.

The community level physiological profile of the biofilm was monitored using Biolog® Ecoplate after 48 and 144 h of exposure to the different concentrations of GBMs. As indicated by the normalized AWCD values as well as the PCA results, a dose-dependent increase of the overall utilization of carbon sources was noticed after 48 h of exposure to GO while the substrate utilization did not differ from the control group after exposure to rGO (Figure 3A; Supplementary Figures S2A,B). In the case of GO exposure, this increase of normalized AWCD values resulted from an increase of the utilization of all the different guilds of carbon sources (Supplementary Figure S3A). At T_144h_, the normalized AWCD values were significantly lower in the control group compared to T_48h_ (*t*-test, *p* = 0.016) and values were not significantly different from the control group after exposure to GBMs (Figure 3B). However, according to PCA results, carbon sources utilization appears to be different from the control in every GO-containing condition (Supplementary Figure S2C). This is associated to an increased utilization of the polymers guild (Supplementary Figure S3B). Similarly, PCA results suggest changes in the CLPP after 144 h of exposure to rGO at 10 mg.L^−1^ compared to unexposed biofilm (Supplementary Figure S2D). In this condition, carbon sources 4-hydroxy benzoic acid, itaconic acid, and D-xylose appeared to be utilized by the microbial consortium while it was not the case in other conditions. The highest average AWCD values measured in the control group at both 48 and 144 h was for the carbohydrate and amino acid guilds while after 144 h of exposure to GO at 1 and 10 mg.L^−1^, the utilization of the substrates from the polymers guild is favored.

### Effects on Bacterial Community Structure

**Figure 4:**
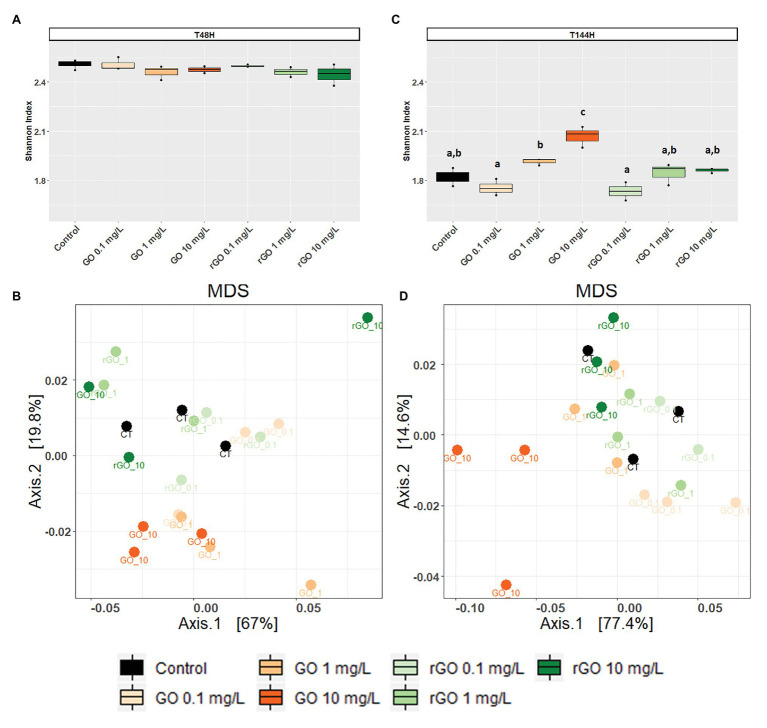
Effects of exposure to GBMs on bacterial communities from the biofilm. Shannon evenness index following 48 h **(A)** or 144 h of exposure to GBMs **(C)** are compared between the exposure conditions. ANOVA followed by Tukey test. Letters indicate significant differences between the tested conditions. Multidimensional scaling (MDS) plot of bacterial communities based on unweighted Unifrac distances after 48 h **(B)** and 144 h **(D)** of exposure to the different conditions.

After 48 h of exposure, the Shannon indexes calculated for the bacterial communities were similar in the GBMs containing conditions compared to the control group (ANOVA *p* = 0.305; Figure 4A) and the bacterial community structure was not significantly affected as revealed by MDS and PERMANOVA analysis using weighted UniFrac distances (PERMANOVA, *p* = 0.221; Figure 4B). Between 48 and 144 h, the trajectory of bacterial communities in control conditions resulted in a decrease of Shannon index (Figure 4B). However, after 144 h of exposure to GO at 10 mg.L^−1^, the Shannon index was significantly higher than in the control (ANOVA, *p* < 0.001; Figure 4C) when compared to the control group. At T_144h_, the exposure to GBMs significantly affected the bacterial community structure (PERMANOVA: *p* = 0.001) and MDS analysis revealed that the effects were more marked after exposure to GO at 10 mg.L^−1^ (Figure 4D).

**Figure 5:**
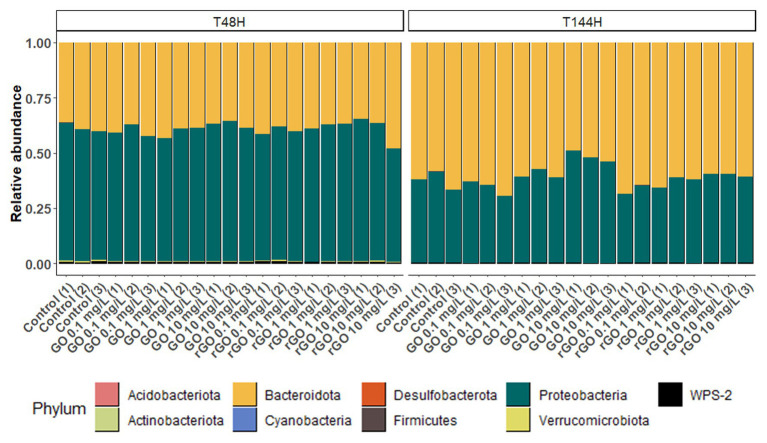
Relative abundance of bacterial phyla from the biofilm after 48 h (T_48h_) and 144 h (T_144h_) of exposure to GO or rGO at concentrations ranging from 0 to 10 mg.L^−1^.

At T_48h_ in the control group, over 98% of the bacteria constituting the biofilm belonged to the phyla Proteobacteria and Bacteroidota, accounting for 60.7 ± 2.0% and 38 ± 1.5% of the total bacteria, respectively (Figure 5). At T_144h_, these two phyla represented over 99% of the whole community but the relative abundance of the phylum Proteobacteria decreased to 37.0 ± 4.9% while it increased to 62.5 ± 4.7% for the phylum Bacteroidota (Figure 5). At T_48h_, the relative abundances of these two phyla were not affected by the GBMs exposure (Bacteroidota: ANOVA, *p* = 0.954; Proteobacteria: ANOVA, *p* = 0.944). After 144 h of exposure to the different concentrations of GBMs, only the exposure to GO at 10 mg.L^−1^ led to significant changes in the phyla distribution. In this condition, the relative abundance of the phylum Bacteroidota was significantly lower (51.8 ± 3.5%) compared to the one observed in the control group (ANOVA, *p* = 0.001 followed by Tuckey test), while the relative abundance of the phylum Proteobacteria was significantly higher (47.8 ± 3.4%; ANOVA, *p* = 0.002 followed by Tuckey test).

**Figure 6:**
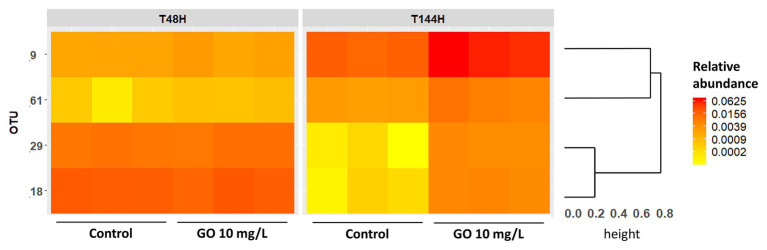
Heatmap showing the relative abundances of the discriminant OTUs identified by Deseq analysis. The dendrogram is based on Bray-Curtis distances metric and hierarchical clustering of OTUs using the complete method.

At T_48h_, OTUs assigned to the phylum Bacteroidota were mainly members of the orders *Chitinophagales* and *Sphingobacteriales* (Supplementary Figure S4A). Members from the order *Burkholderiales* and *Rhodobacterales* predominated among OTUs affiliated to the phylum Proteobacteria (Supplementary Figure S4B). While *Burkholderiales* and *Rhodobacterales* members still predominated over other Proteobacteria after 144 h of incubation, the relative abundance of OTUs affiliated to the Order *Xanthomonadales* increased over time in all conditions (Supplementary Figure S4B). Statistical analysis of the OTUs relative abundances in the biofilm indicated that four OTUs from the phylum Proteobacteria were differentially abundant upon exposure to GBMs, with a significance threshold fixed at *p* = 0.01. All four OTUs were more abundant after exposure to 10 mg.L^−1^ of GO during 144 h than in control condition. Two of these OTUs, with a log2-fold change of 6.79 ± 1.2 and 5.82 ± 1.1, were affiliated to the genus *Acidovorax* while the two others with a log2-fold change of 1.81 ± 0.4 and 1.95 ± 0.2 belonged to the genus *Silanimonas* (Table 2). In the control group, the relative abundance of the two *Acidovorax* members (OTUs 29 and 18) decreased between T_48h_ and T_144h_ while it increased for the two *Silanimonas* sp. (OTUs 9 and 61; Figure 6). Upon exposure to GO at 10 mg.L^−1^, these OTUs followed different trajectories compared to the control conditions. Thus, the increases of the former OTUs were enhanced while the decreases were less marked over time for the latter OTUs (Figure 6). For example, the OTU 9 increased from 0.18 ± 0.02% at T_48h_ to 1.7 ± 0.2% at T_144h_ in the control group whereas it reached 6.2 ± 1.2% following exposure to GO at 10 mg.L^−1^.

**Table 2:** Operational taxonomic units (OTUs) differentially abundant (*p* < 0.01) at 10 mg.L^−1^ of GO compared to the control group after 144 h of exposure.

OTU	Log2-fold change	Phylum	Class	Order	Family	Genus
18	6.79	Proteobacteria	*Gammaproteobacteria*	*Burkholderiales*	*Comamonadaceae*	*Acidovorax*
29	5.82	Proteobacteria	*Gammaproteobacteria*	*Burkholderiales*	*Comamonadaceae*	*Acidovorax*
61	1.81	Proteobacteria	*Gammaproteobacteria*	*Xanthomonadales*	*Xanthomonadaceae*	*Silanimonas*
9	1.95	Proteobacteria	*Gammaproteobacteria*	*Xanthomonadales*	*Xanthomonadaceae*	*Silanimonas*

Positive log2-fold change values indicate enriched OTUs in the exposure condition.

## Discussion

Due to the multiple crucial roles played by microbial communities in the environment, studying the effects of GBMs toward these communities is essential to better assess the ecosystemic consequences of a contamination of the environment by these nanomaterials. For this purpose, many studies evaluated their ecotoxic potential through the use of single-species-based assays. These tests are relevant to determine the toxicological effects and pathways associated to GBMs exposure but they are not realistic and fail to inform the consequences that could occur when interacting species are exposed. As litterature about GBMs impact on complex communities is scarce for the aquatic environment, this study aims to fill the gap on the subject.

### Consequences of the GBMs Exposure on the Algal Compartment

In our study, exposure to the different GBMs concentrations led to a growth stimulation of the diatom *N. palea*, reaching the maximum growth rate earlier compared to the control group, the growth rate of the latter being comparable to previous studies (Garacci et al., 2017). These results are contradictory with the vast majority of single-species-based studies which indicated that exposure to engineered nanoparticles led to algal growth inhibition associated to oxidative stress induced by cellular damages (Chen et al., 2019; Saxena et al., 2020). For example, this was observed for green pelagic algae such as *Raphidocelis subcapitata*, *Chlorella pyrenoidosa*, or *Scenedesmus obliquus* after exposure to GO or rGO (Du et al., 2016; Zhao et al., 2017, 2019; Malina et al., 2019). It was also indicated that the exposure of the freshwater diatom *N. palea* to few layer graphene (FLG) increased the production of EPS substances which mitigated its toxicity (Garacci et al., 2017, 2019). Despite the difference of sensitivity of the different algal species to GBMs, the concentrations leading to 50% of algal growth inhibitions all ranged from 20 to over 150 mg.L^−1^, which is approximately one order of magnitude higher than the concentrations tested in our study (Nogueira et al., 2015; Du et al., 2016; Zhao et al., 2017). In our work, the similar trends observed on the increase of the diatom growth following exposure to GO or rGO suggest that this response may not be influenced by specific physico-chemical characteristics such as oxidation level. In addition, the weak oxidative stress measured in the diatom is consistent with the absence of growth impairment and highlights the protective effect associated to the biofilm lifestyle. Growth inhibitory effects were also hypothesized to be associated to indirect ecotoxic effects through shading as well as to nutrient depletion by GBMs (Garacci et al., 2017; Zhao et al., 2017). As the algal growth increased, these two hypotheses are unlikely under the exposure conditions used in this study or they could have been counterbalanced by favorable interactions with bacteria.

According to the FCM analyses performed, we observed that exposure to GO led to an increase of chlorophyll content and to a decrease of lipid content in the diatom while opposite results were observed following rGO exposure. Although few studies monitored changes in algal physiological parameters, the results obtained in the latter case are in line with studies from the literature focusing on the effects associated to low-oxygen content GBMs exposure. Indeed, a significant reduction of chlorophyll level and an under-expression of genes associated to chlorophyll biosynthesis resulted from an exposure to rGO or few layer graphene in the algae *Scenedesmus obliquus* or *N. palea*, respectively (Du et al., 2016; Garacci et al., 2019). In addition, exposure to graphene nanosheets was shown to favor lipid accumulation in *Chlorella pyrenoidosa* (Khanra et al., 2018). However, in the case of GO exposure, our results are contradictory with the single-species-based literature that usually reports a decrease of chlorophyll pigments (Tang et al., 2015; Hu et al., 2016; Hazeem et al., 2017; Malina et al., 2019). *Nitzschia palea* is a mixotrophic diatom able to grow using autotrophic metabolism (e.g., photosynthesis) using an inorganic carbon source (CO_2_) and/or through a heterotrophic metabolism using organic carbon sources (Villanova et al., 2017). It was reported that mixotrophic algae accumulated chlorophyll when cultured in autotrophy, while this content decreased and lipids were accumulated by the algae under heterotrophic conditions (Cheirsilp and Torpee, 2012; da Silva Ferreira and Sant’Anna, 2017). Thus, our observations suggest that in *N. palea*, when it is associated to a bacterial consortium, autotrophic and heterotrophic metabolism were differentially balanced under GO or rGO exposure. In any case, these changes of the energy acquisition pathways in the diatom did not alter growth performance following 144 h of exposure to GBMs.

### Consequences of the GBMs Exposure on the Bacterial Compartment

The effects of GBMs toward bacteria have been mainly investigated through the exposure of single bacterial strains. The effects toward bacterial communities were studied in soil (Du et al., 2015; Xiong et al., 2018; Forstner et al., 2019) or in activated sludge (Ahmed and Rodrigues, 2013; Guo et al., 2018; Yujie et al., 2020) but data remain scarce in aquatic ecosystems (Evariste et al., 2020). Given the important bacterial growth inhibition and the relatively weak consequences on microbial composition noticed after 48 h of exposure to GO, we can suggest that the effects of this nanomaterial could be associated to a mechanism that impacts in a similar manner most bacterial species found in this biofilm. Previous studies suggested that the membrane composition, especially in gram-negative bacteria, as well as bacterial shape could constitute a criteria of resistance to GBMs (Kang et al., 2009; Akhavan and Ghaderi, 2010; Pulingam et al., 2019; Sengupta et al., 2019). Despite that the four OTUs benefiting from exposure to high GO concentrations are Gram-negative bacteria, it is more likely that their metabolic capacities are involved in the greater tolerance to GO. Indeed, it was recently reported that bacteria from the genus *Acidovorax* sp. were tolerant to GO concentrations up to 95 mg.L^−1^ in granulated sludge treating wastewater (Kedves et al., 2020). This genus is also well-known to be able to degrade organic matter in wastewater treatment plants (Schulze et al., 1999) as well as aromatical compounds like phenanthrene (Singleton et al., 2018) or biphenyl (Ohtsubo et al., 2012). Similarly, *Silanimonas* spp. are categoricized as benzene-degrading species (Mosmeri et al., 2019). Thus, we can hypothesize that these graphene-tolerant bacteria benefited from the presence of GBMs in the media (i) by outcompeting other less tolerant species and (ii) because they could be able to degrade or modify the GO structure.

According to the literature, bactericidal activities of GO and rGO toward planktonic bacteria indicated generally stronger bactericidal effects of GO compared to rGO as observed in our study (Han et al., 2019). However, results concerning antibacterial effects toward bacterial biofilms are more contradictory. Indeed, strong bactericidal effects of GO were previously observed like in our study (Mejías Carpio et al., 2012; Yadav et al., 2017; Giulio et al., 2018; Pandit et al., 2018; Song et al., 2018) while others indicated that GO enhanced biofilm formation and that rGO exerted strong inhibitory effects (Ruiz et al., 2011; Guo et al., 2017). The interstudy discrepancies may be associated to several causes influencing the biological responses. Among the previously cited studies, when available, the characterization data indicate a wide variability within the range of oxygen content of the tested GO (from 27.8 to over 36 atomic %), with different distribution of the types of oxygen-containing functions. In addition, depending on the method applied, the reduction of these materials lead to the production of a wide variety of rGO. This underline the need to provide detailed characterization data and the use of a classification framework to facilitate interstudy comparisons (Wick et al., 2014). Among other possible causes, it was indicated that the effects of GBMs may vary depending on the medium composition and/or on the biofilm maturity (Hui et al., 2014; Guo et al., 2017; Fallatah et al., 2019). The results obtained in our study are in line with the latter assumption as the IC_50_ values increased between T_48h_ and T_144h_, indicating that the effects of GO on bacterial growth were mitigated over time with biofilm maturation. This suggests that bacterial growth was delayed by GO exposure and that bacterial communities were recovering. However, after 144 h of exposure, the presence of GBMs in the media resulted in bacterial communities with diverging community structure trajectories compared to the control group. Further research would be needed to determine if bacterial communities are able to fully recover and to assess the time duration necessary for full recovery.

In addition to the effects on bacterial growth and diversity, exposure to GBMs was shown to influence microbial activities and could potentially lead to disturbances of carbon or nitrogen cycles (Zhou et al., 2019; Yilihamu et al., 2020). For example, previous studies indicated that exposure to GO or rGO could enhance the anaerobic ammonium oxidation activity that is involved in nitrogen removal in ecosystems (Wang et al., 2013; Yin et al., 2015; Tomaszewski et al., 2019). Extracellular enymatic activities involved in carbon cycling were also shown to be lowered by short term exposure to GO (Chung et al., 2015). The results obtained in our study using Biolog Ecoplates suggested that the effects of GBMs exposure on the community level physiological profiles were transitory. Indeed, the activity levels of the microbial communities were similar to the control group in the different tested conditions after 144 h of exposure. However, this method does not allow to determine if the increased activity is associated to an increase of intra- or extracellular metabolism. The recovery of carbon-related metabolic activities despite the changes occuring in microbial community composition could be explained by functional redundancy between the species composing the biofilm. Further experiment using other omics tools would be needed to examine the metabolic pathways that are influenced by GBMs exposure and to determine if the metabolism of compounds involved in algae-bacteria mutualistic interactions are modified (Cooper and Smith, 2015).

### Consequences of the Diatom-Bacteria Interactions on the Response to GBMs Exposure

In the case of mixed-species biofilms containing phototropic microorganisms like diatoms as in our study case, the EPS produced can be used by heterotrophic bacteria as carbon and energy sources (Mühlenbruch et al., 2018). In return, bacteria may benefit diatoms by providing sources of nutrients such as vitamins or nitrogen but are also able to reduce algal oxidative stress through the production of enzymes such as catalase (Hünken et al., 2008; Amin et al., 2012; Natrah et al., 2014). Diatom-associated microbiota from environmental samples was described to mainly belong to the phylum Proteobacteria and Bacteroidota (*ex* phylum Bacteroidetes) that are involved in EPS degradations (Schäfer et al., 2002; Amin et al., 2012; Bohórquez et al., 2017). Thus, the composition of the bacterial compartment in the biofilm of our study is consistent with the data from the literature. The heterotrophic bacteria remineralize the organic matter produced by the diatom, ensuring an efficient nutrient cycling (Christie-Oleza et al., 2017). The functioning of biofilms relies on this mutual benefit between the algae and the bacterial consortium. In the present study, diatom and bacteria growth were not correlated across our experimental conditions, demonstrating an interplay between multiple/complex interactions and GBMs direct effects.

It has been previously shown that the exposure to carbon-based nanomaterials such as carbon nanotubes and FLG increased the EPS excretion by *N. palea* (Verneuil et al., 2015; Garacci et al., 2017). This mechanism was suggested to constitute a strategy allowing toxicity mitigation for the algae. However, this could have several consequences for the bacterial compartment. Indeed, the embedment of GBMs into the biofilm EPS could increase contacts with bacteria and favor membrane impairments (Dizaj et al., 2015), which could explain the strong bacterial growth inhibition that we observed in presence of GO. The differential effects observed between GO and rGO on bacterial growth might be associated to the oxidation level of the materials. Indeed, the GBMs were shown to be able to interact with natural organic matter including polysaccharydes (Chowdhury et al., 2014). As these interactions depend on the nature of oxygen-containg functional groups (Saya et al., 2021), the interaction dynamics between EPS and GO or rGO could be different, influencing the contact with bacterial cells embeded in EPS. In addition to the increase in EPS excretion, the composition and the quality of the EPS produced by the diatoms could be modified by the presence of GBMs. This was previously observed after exposure of bacteria or algae to metallic nanoparticles (Hou et al., 2015, 2017; You et al., 2015; Chen et al., 2019). Moreover, it was suggested that the energy balance between growth and EPS production could be modified following exposure to nanoparticles that is likely to occur in the case of the growth stimulation observed in our study (Taylor et al., 2016). As it was indicated that the EPS composition could affect the bacterial communities in biofilms (Haynes et al., 2007; Bohórquez et al., 2017), we can hypothesize that the changes observed in the bacterial communities could be associated to indirect effects associated to modifications of the EPS quantity and quality.

As discussed in paragraph “Consequences of the GBMs Exposure on the Algal Compartment,” the growth stimulation observed in the diatom is more likely to be due to indirect effects. We can hypothesize that the overproduction of EPS associated to the presence of GBMs might benefit *in fine* to the diatoms through the increase of metabolite quantities produced by bacterial activity. In addition, the results obtained from the community level physiological profiles indicated an activation of heterotrophic metabolism of the biofilm following short term exposure to GO. This metabolic increase allowed to compensate the loss of activity that can be expected due to the bacterial growth inhibition measured in presence of GO. Indeed, considering the AWCD values for the whole biofilm (data not shown) instead of AWCD per bacterial cell as previously presented, the overall carbon-related metabolic activities are maintained at the level of the control group until 1 mg.L^−1^ and a slight decrease is noticed at 10 mg.L^−1^ of GO. This should allow to maintain a baseline carbon and nutrient cycling between the algae and the bacteria.

## Conclusion

In this study, we observed that the biological responses of mixed microbial communities facing exposure to GBMs were complex and contradictory to the results of single-species experiments found in the litterature. Here and according to the obtained data, we suggest that the biofilm responses were mainly associated to indirect effects initiated by the overproduction of EPS by the diatom. This potentially increased the amount of carbon available for bacterial metabolism and further benefit to the diatom. However, the oxidized form of graphene was shown to strongly impact bacterial biomass. The activities of carbon sources utilization were maintained in most conditions except at 10 mg.L^−1^ of GO that is unlikely to occur under a scenario of chronic release into the environment. However, changes associated to the diatom physiology and changes in bacterial community composition might lead to modifications of the biofilm biochemical properties and potentially affect higher trophic level organisms feeding on it. Thus, future research on the environmental risk associated to GBMs should increase environmental relevance of the bioassays.

## Data Availability Statement

The original contributions presented in the study are publicly available. These data can be found in NCBI under accession number PRJNA674395.

## Author Contributions

LE: writing original draft – investigation – formal analysis. PB: formal analysis – investigation. FM and LG: project administration – funding acquisition. JS: resources – methodology. EF: formal analysis – funding acquisition – review and editing. EP: supervision – review and editing. MB: writing, review and editing – methodology – supervision. All authors contributed to the article and approved the submitted version.

### Conflict of Interest

The authors declare that the research was conducted in the absence of any commercial or financial relationships that could be construed as a potential conflict of interest.
