# Supplementary material for: Graphene-Based Nanomaterials Modulate Internal Biofilm Interactions and Microbial Diversity
Source: Front Microbiol. 2021 Mar 26;12:623853. doi: 10.3389/fmicb.2021.623853 (PMC8032548; doi:10.3389/fmicb.2021.623853)
Supplement: Supplementary file 1 [file Table_1.docx]

**Graphene-based nanomaterials modulate internal biofilm interactions and microbial diversity**

**Supplementary data**

Lauris Evariste^1^, Paul Braylé^1^, Florence Mouchet^1^, Jérôme Silvestre^1^, Laury Gauthier^1^, Emmanuel Flahaut^2^, Eric Pinelli^1^, Maialen Barret^1^

^1^Laboratoire d’écologie fonctionnelle et environnement, Université de Toulouse, CNRS, INPT, UPS, Toulouse, France

^2^CIRIMAT, Université de Toulouse, CNRS, INPT, UPS, UMR CNRS-UPS-INP N°5085, Université Toulouse 3 Paul Sabatier, Bât. CIRIMAT, 118 route de Narbonne, 31062 Toulouse cedex 9, France


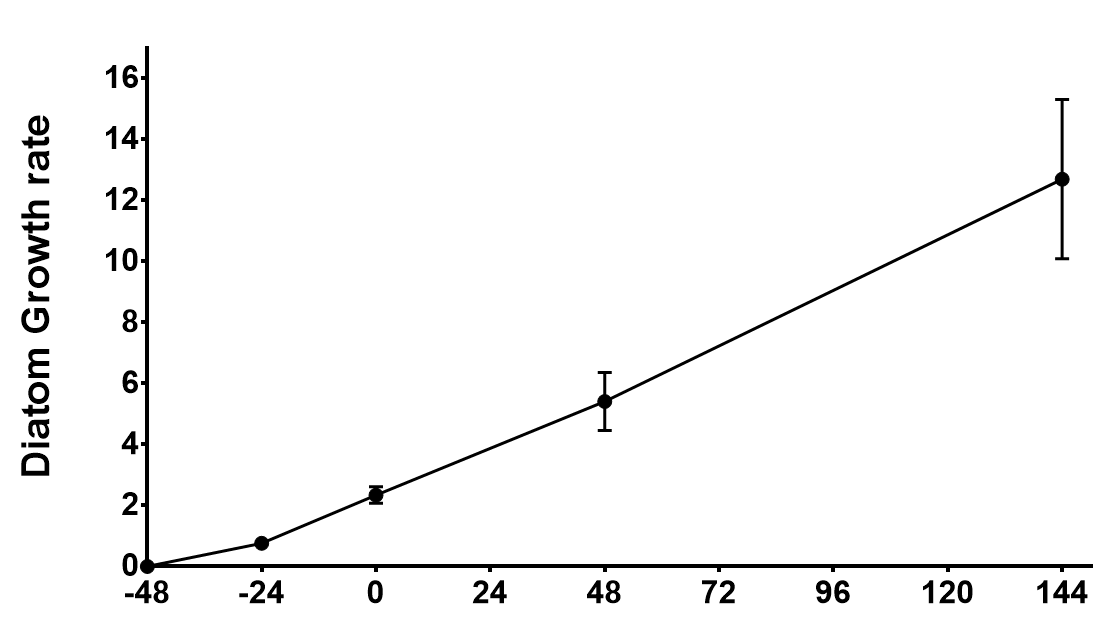


Figure S1: Growth rate curve of *N. palea* in control conditions over the experiment duration. Diatoms counting was carried out using flow cytometry. Results are presented as mean ± standard error of three replicates.


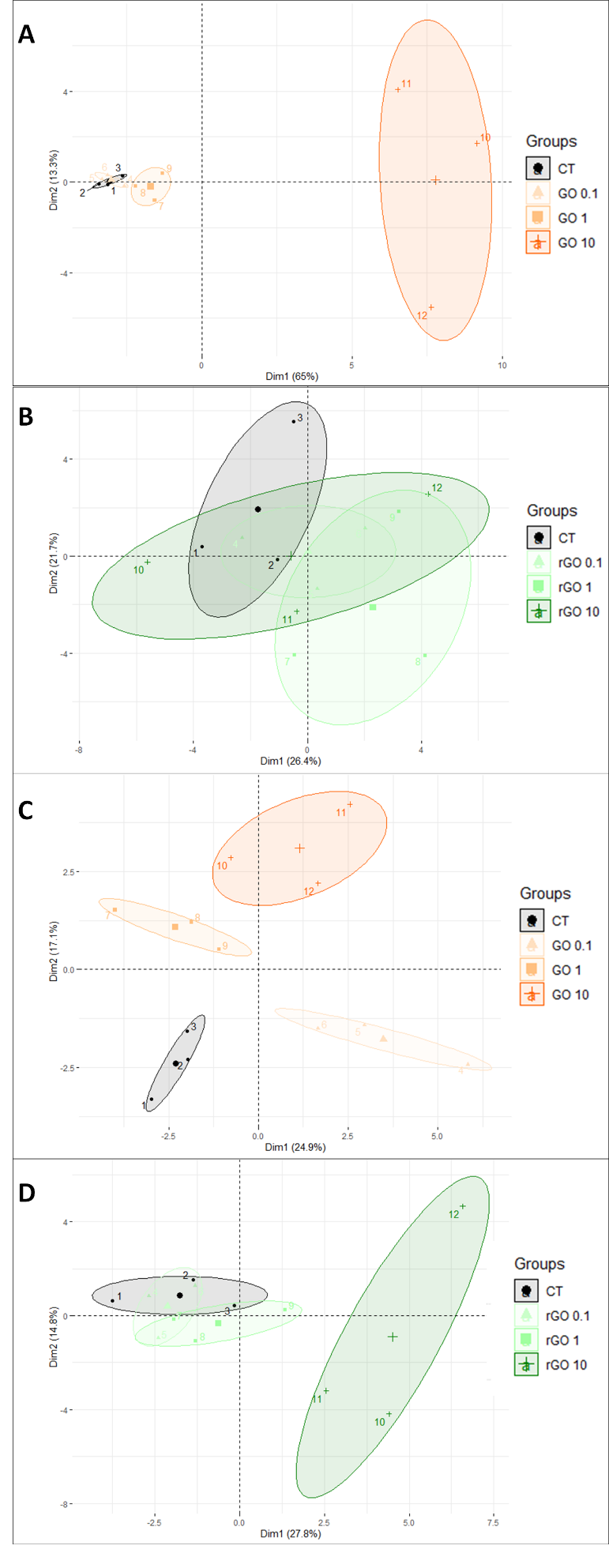


Figure S2: PCA of normalized AWCD data following 48 hours of exposure to GO (A) or rGO (B) and after 144 hours of exposure to GO (C) and rGO (D).


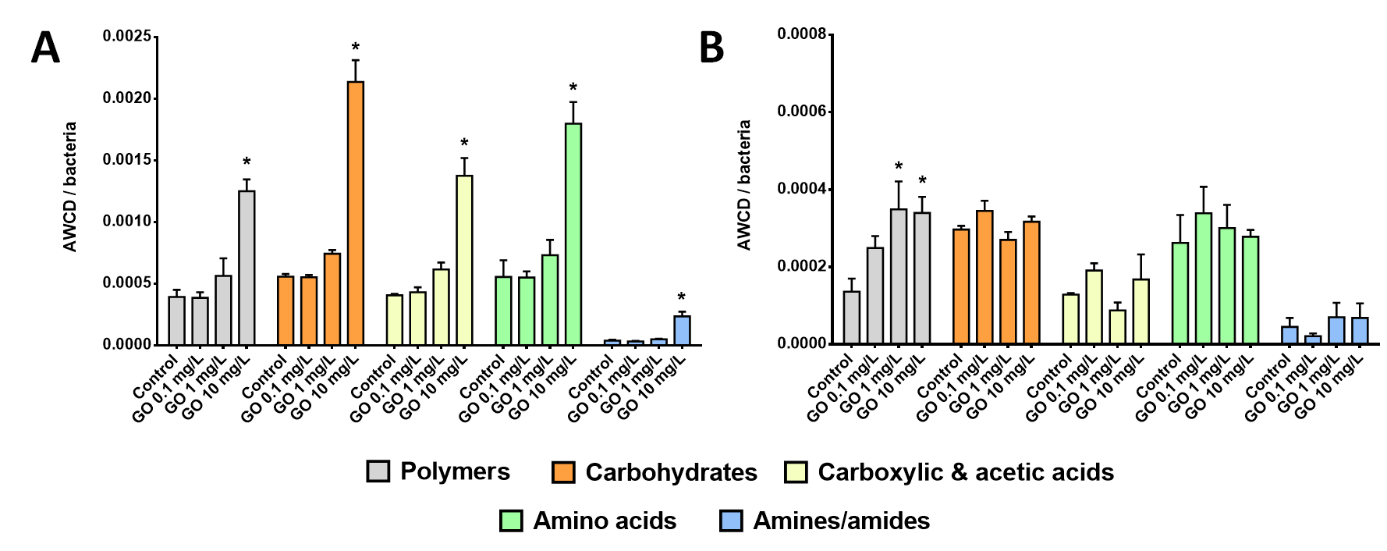


Figure S3: AWCD values measured for each guild of carbon sources after 48 hours (A) and 144 hours (B) of exposure to GBMs. ANOVA (*p* < 0.05) was followed by Tukey test. Asterisks indicate groups significantly different from the control group.


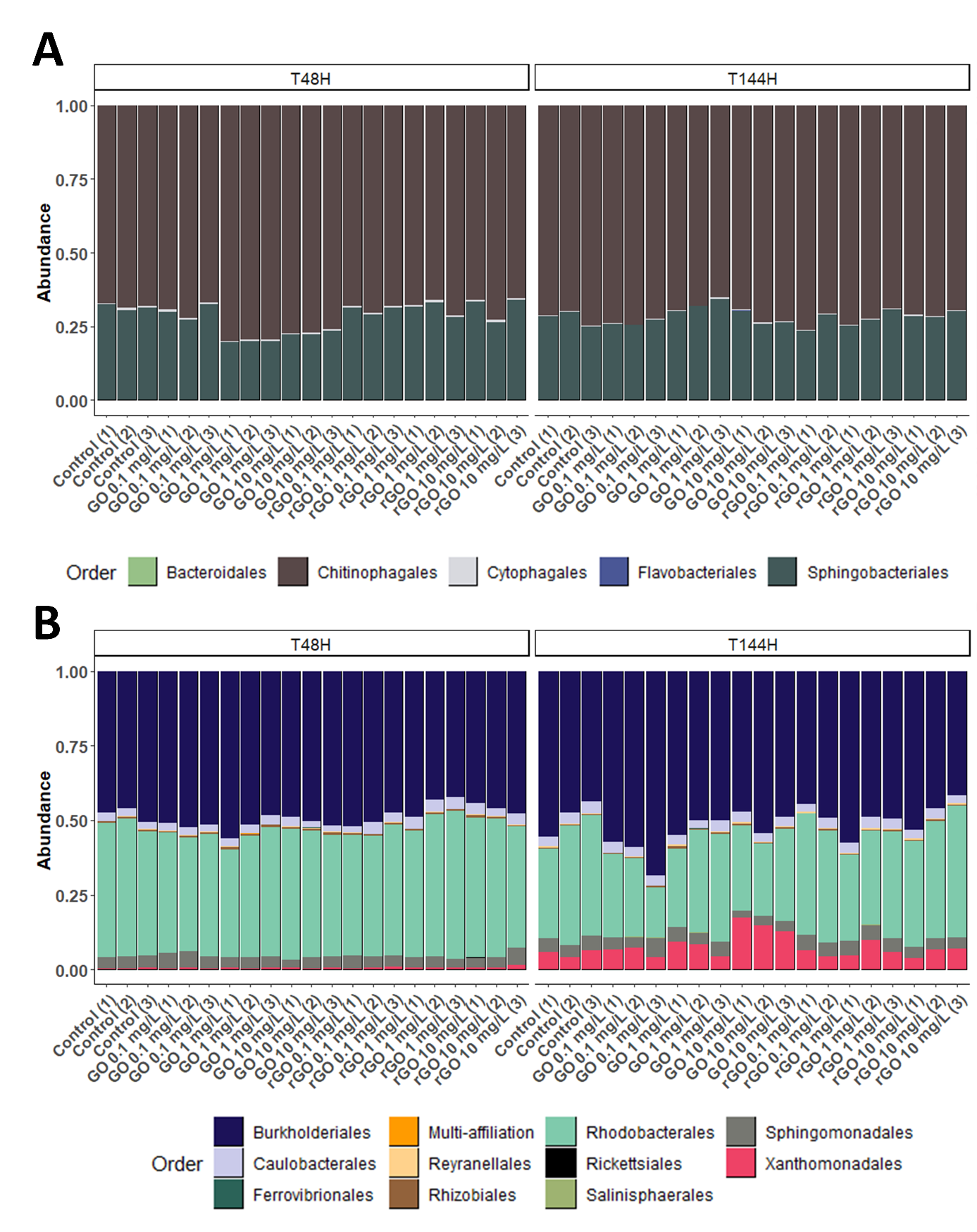
Figure S4: Relative abundance of bacterial Order from the phyla Bacteroidota (A) and Proteobacteria (B) following exposure to GBMs concentrations ranging from 0 to 10 mg/L.
